# The MDA-MB-231 Breast Cancer Cell Secretomes Modify Metabolomes of *Pseudomonas aeruginosa* Breast Microbiome

**DOI:** 10.3390/ijms26115003

**Published:** 2025-05-22

**Authors:** Majdoleen AlDawsari, Mysoon M. Al-Ansari, Reem H. AlMalki, Anas M. Abdel Rahman, Monther Al-Alwan

**Affiliations:** 1 Department of Botany and Microbiology, College of Science, King Saud University, Riyadh 11451, Saudi Arabia; majdoleenaldawsari@hotmail.com; 2 Cell Therapy and Immunobiology Department, Research and Innovation, King Faisal Specialist Hospital and Research Center, Riyadh 11211, Saudi Arabia; 3 Metabolomics Section, Precision Medicine Laboratory Department, Genomics Medicine Center of Excellence, King Faisal Specialist Hospital and Research Center, Riyadh 11211, Saudi Arabia; rgalmalki@kfshrc.edu.sa (R.H.A.); aabdelrahman46@kfshrc.edu.sa (A.M.A.R.); 4 Department of Biochemistry and Molecular Medicine, Al-Faisal University, Riyadh 11533, Saudi Arabia; 5 College of Medicine, Al-Faisal University, Riyadh 11533, Saudi Arabia

**Keywords:** breast cancer, metabolic pathways, untargeted metabolomics, microbiome

## Abstract

Breast cancer (BC) is globally becoming a great challenge, being both the most diagnosed cancer and the leading cause of death in women. In addition to cancer cells, many bacteria co-inhabit BC, which differ in type and number from the resident microbiota found in healthy breast tissue. While many reports have demonstrated the ability of different bacteria to dysregulate BC’s metabolites, the reciprocal effect of these metabolites on the bacterial microbiota has not yet been investigated. Herein, we assess the effect of conditioned media (CM) from a triple-negative BC cell line (MDA-MB-231) on the metabolic profile of *Pseudomonas aeruginosa* (*P. aeruginosa*), an important breast resident Gram-negative bacteria that influence oncogenesis. Optical density and scanning electron microscopes were used to assess the impact of MDA-MB-231-CM (BC-CM) on *P. aeruginosa* growth and morphological changes, respectively. In addition, liquid chromatography–high-resolution mass spectrometry was used to identify metabolic changes in *P. aeruginosa* and their secretomes in response to the BC-CM. The BC-CM significantly suppressed the growth of *P. aeruginosa* in the log phase and induced concentration-dependent cytopathological changes in their cell walls. The metabolites of *P. aeruginosa* were dysregulated considerably depending on the time of exposure to the BC-CM. When treated with the BC-CM, *P. aeruginosa* induced the purine alkaloid spliceostatin (FR901464), a prominent antitumor metabolite. The BC-CM also promoted other *P. aeruginosa* metabolites such as amino acids, phosphoribosyl-AMP, 2-aminoacetophenone, pyochelin I, guanosine monophosphate, riboflavin, and terpenoids, which are capable of interfering with oncogenesis. Nine of the significantly identified metabolites from the 0–3 h comparison and four of those identified from the 0–6 h comparison have potential roles in influencing cancer cell behavior. Our findings demonstrate the ability of triple-negative BC-CM not only to alter the growth and morphology of *P. aeruginosa* but also to modulate their metabolic profile. A better understanding of the influence of BC on certain resident breast microbiomes, such as *P. aeruginosa,* may open a new therapeutic intervention opportunity for the treatment of cancer.

## 1. Introduction

Breast cancer (BC) is emerging as a challenging health issue, affecting 2.3 million cases each year and causing 670,000 deaths globally in 2022. In the US alone, it is expected that nearly 13% (about one in eight) of women will develop invasive BC during their lifetime [1]. Numerous risk factors contribute to BC oncogenesis, including age, estrogen levels, hormone imbalances, lifestyle changes, contraceptive medications, genetic mutations, and metabolic changes in the breast [2,3]. Recent studies have revealed that the breast tissues are colonized with various bacteria and function as commensals, despite the mammary gland’s putative sterility. The tumor’s microenvironment is also home to a variety of microorganisms in addition to cancer cells. 16S rRNA sequencing revealed variable types of bacteria, including *Pseudomonas*, *Bacillus*, *Prevotella*, *Acinetobacter*, *Listeria*, *Lactobacillus*, and members of the Enterobacteriaceae class, in healthy breast tissue [4]. Previous studies have shown that microbiota can enter breast tissue through the nipple’s ducts or via internal circulation from the gut [5,6,7]. Some bacteria have been shown to invade tumors, in some cases suppressing tumor growth, while specific pathogens have been reported to contribute to cancer development through mechanisms like persistent infection, immune evasion, and chronic inflammation [6].

Metabolites of the breast microbiome interact with estrogen functions and support antitumor activity and immune surveillance [8,9]. An excess of free circulating estrogen is a risk factor for breast tumorigenesis, especially in postmenopausal women. The estrobolome, a collection of enteric bacteria involved in estrogen metabolism, influences free estrogen levels, with the gut microbiome diversity and composition influencing estrogen metabolism [10]. Moreover, the microbiota can act as a key player in altering the metabolism within the breast microenvironment and induce malignancies [11]. Researchers have demonstrated that the resident microbial communities contribute to breast health by degrading potent carcinogens and activating immune surveillance pathways [12]. Among the different breast microbiota, *P. aeruginosa*, a Gram-negative bacterium, accounts for 27% of the reported cases in breast microbiota, which produces pyocyanin metabolites that modulate host immunity [13,14]. Approximately 90–95% of *P. aeruginosa* produce the pigment pyocyanin, which has strong antibacterial, antioxidant, and anticancer effects [15]. The *P. aeruginosa* makes mannose-sensitive hemagglutinin (PA-MSHA), which is very effective against BC cell proliferation [16], and synthesized the metabolite azurin, which has been proven to be an anti-BC agent [17]. Studies have reported variation in bacterial strains depending on the molecular subtype of BC, the grade, or the aggressiveness of the disease [18]. Inflammation induced by certain bacteria has been linked to increased estrogen release, a known risk factor for BC [19]. Moreover, intracellular microbiomes can rearrange cancer cells’ exoskeleton and facilitate resistance development [16].

A previous study demonstrated the ability of *Escherichia coli* to alter the metabolomic profile of MDA-MB-231 triple-negative BC cells [20]. The oncobiome in the BC sites was reported to complicate treatment options like chemo- and radiotherapy, as well as to modulate the immune response against cancer antigens [21]. Accordingly, we hypothesized that BC cell secretomes can influence the metabolomic profile of bacterial microbiota. This study was designed to investigate important ulstered metabolites that have the potential to influence treatment, inflammation, or cancer activity according to the cancer environment. Therefore, this study examined the effect of secretomes released by BC cells (MDA-MB-231) on the metabolomic profile of a commensal bacteria, *P. aeruginosa*, a relevant and valuable model for exploring host–microbe interactions in breast cancer. *P. aeruginosa* is one of the most abundant genera in breast tumor tissues, suggesting a potential role in tumor-associated microbial dynamics [22]. Additionally, *P. aeruginosa* has been shown to produce metabolites like azurin, which can modulate host immunity and even suppress breast cancer cell proliferation [23]. Its relative resistance to chemotherapy and increased abundance following treatment further indicate its clinical significance in the tumor microenvironment [24]. Moreover, recent findings suggest that bacterial signals, including those from *P. aeruginosa*, may contribute to cancer progression or treatment resistance, highlighting the importance of studying their metabolomic interactions with cancer cells [25]. The outcome of investigating the impact of BC on the metabolomic profiles of *P. aeruginosa* can provide useful insight and open new avenues for potential therapeutic targeting.

## 2. Results

### 2.1. Breast Cancer-Conditioned Media Suppress the Growth Rate of Pseudomonas aeruginosa

**Figure 1:**
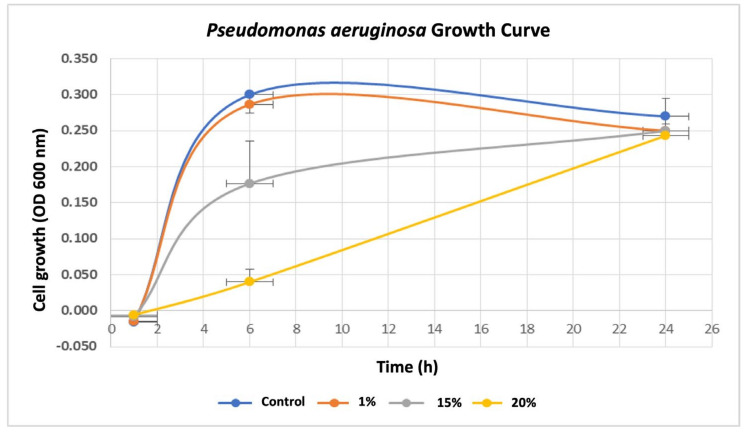
Growth rate of *P. aeruginosa* treated with breast cancer-conditioned media. *P. aeruginosa* was treated with different concentrations (1, 15, and 20%) of MDA-MB-231 cell-conditioned media or serum-free conditioned media (0% control). Treated *P. aeruginosa* was collected at different time points (1, 6, and 24 h), and their growth rates were assessed by measuring optical density (OD) at 600 nm. The results are expressed as the means ± standard deviations of 3 replicates.

To investigate the influence of breast cancer cells on *P. aeruginosa*, we first assessed the effects of various concentrations (0%, 1%, 15%, and 20%) of breast cancer-conditioned media (BC-CM) derived from MDA-MB-231 cells on the growth rate of *P. aeruginosa* by measuring the optical density (OD) at various time points (0, 1, 6, and 24 h). Our results showed that *P. aeruginosa* treated with 1% BC-CM reached its log phase after 6 h of incubation, exhibiting a parallel growth with no significant differences (*p* value = 0.116) when compared to the control, which were treated with cell-free CM (Figure 1). Similarly, *P. aeruginosa* treated with 1% BC-CM showed no significant differences (*p* value = 0.184) at the decline (24 h) phase when compared to the control. Interestingly, *P. aeruginosa* treated with 15% and 20% BC-CM showed a dose-dependent significant growth inhibition when compared to the control at 6 h (*p*-value = 0.0218 and 0.0014, respectively). However, the growth of *P. aeruginosa* treated with 15% and 20% BC-CM showed no statistical significance (*p.* value = 0.269 and 0.635, respectively) when compared to the control at 24 h. Collectively, these results suggest that the inhibitory effect of high concentrations of BC-CM at the early growth stages of *P. aeruginosa* may help in creating more adaptive and persistent types of bacterial cells that are able to reproduce for a longer period.

### 2.2. Breast Cancer-Conditioned Media Alter P. aeruginosa Cell Morphology

**Figure 2:**
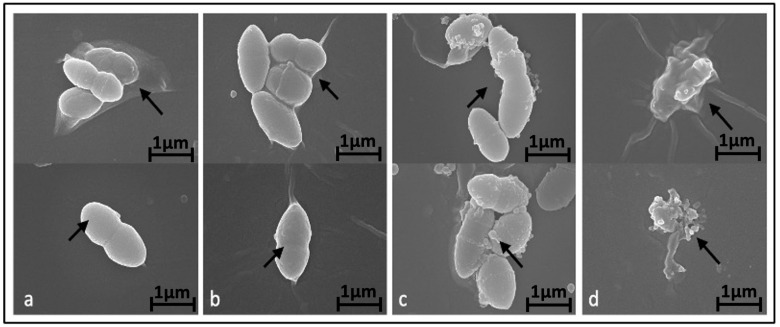
Alteration of *P. aeruginosa* morphology treated with breast cancer-conditioned media. Scanning electron microscopy representative images showing morphology of *P. aeruginosa* treated for 6 h with either 0% (**a**), 1% (**b**), 15% (**c**), or 20% (**d**) of MDA-MB-231 cell-conditioned media. Arrows indicate extracellular morphological changes with increased doses of conditioned media. Magnification of the displayed images is X 25,000.

To investigate the potential impact of BC-CM on bacterial morphology, we examined the effect of various concentrations of BC-CM on *P. aeruginosa* cells after 6 h of incubation using scanning electron microscopy (SEM). Our results indicated that *P. aeruginosa* cells treated with 1% BC-CM did not exhibit any significant morphological changes compared to the control, maintaining well-intact cell walls and membranes (Figure 2a,b). In contrast, *P. aeruginosa* cells treated with higher concentrations of BC-CM (15% and 20%) exhibited altered cell morphology and biofilm architecture (Figure 2c,d). Moreover, the degree of bacterial morphological changes was pronounced in the group treated with 20% BC-CM compared to the 15% BC-CM-treated group (Figure 2c,d). These findings indicate that BC-CM have a significant inhibitory effect on *P. aeruginosa*, impairing growth and causing structural distortions and biofilm disruption.

### 2.3. Metabolites Induced by MDA-MB-231-Conditioned Media

**Figure 3:**
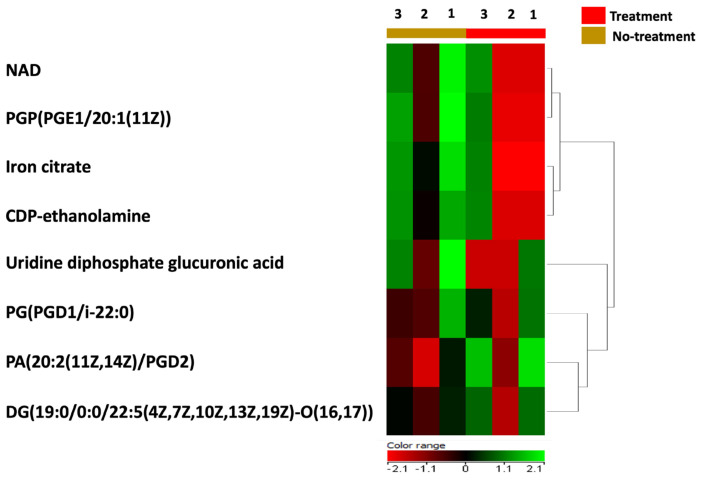
A heatmap representing the upregulated metabolites in BC-CM after mixing with *P. aeruginosa*. The heatmap shows replicates of dysregulated metabolites in BC-CM once mixed for 0 h with *P. aeruginosa* (treatment) compared to the serum-free media (no-treatment).

In order to test the direct effect of BC-CM on *P. aeruginosa*, we analyzed and profiled the BC-CM at the first interaction (0 h) with *P. aeruginosa* using the Human Metabolome Database (HMDB). This approach was chosen because the direct analysis of the MDA-MB-231 secretome alone does not reflect the dynamic interactions between cancer secretomes and bacteria. Instead, analyzing the BC-CM after bacterial interaction provides a more comprehensive understanding of these communications, making the study more relevant to real biological environments. The Human Metabolome Database identified 36 BC-CM metabolites mixed with *P. aeruginosa* when compared to the control (SFM). Eight out of thirty-six metabolites that were related to MDA-MB-231 cells, including iron citrate, NAD, CDP-ethanolamine, uridine diphosphate glucuronic acid, PGP(PGE1/20:1(11Z)), PG(PGD1/i22:0), PA(20:2(11Z,14Z)/PGD2), and DG(19:0/0:0/22:5(4Z,7Z,10Z,13Z,19Z)-O(16,17)) (Table S1), were significantly upregulated compared to SFM control (Figure 3).

### 2.4. Breast Cancer-Conditioned Media Disrupts P. aeruginosa Metabolomics Profile

**Figure 4:**
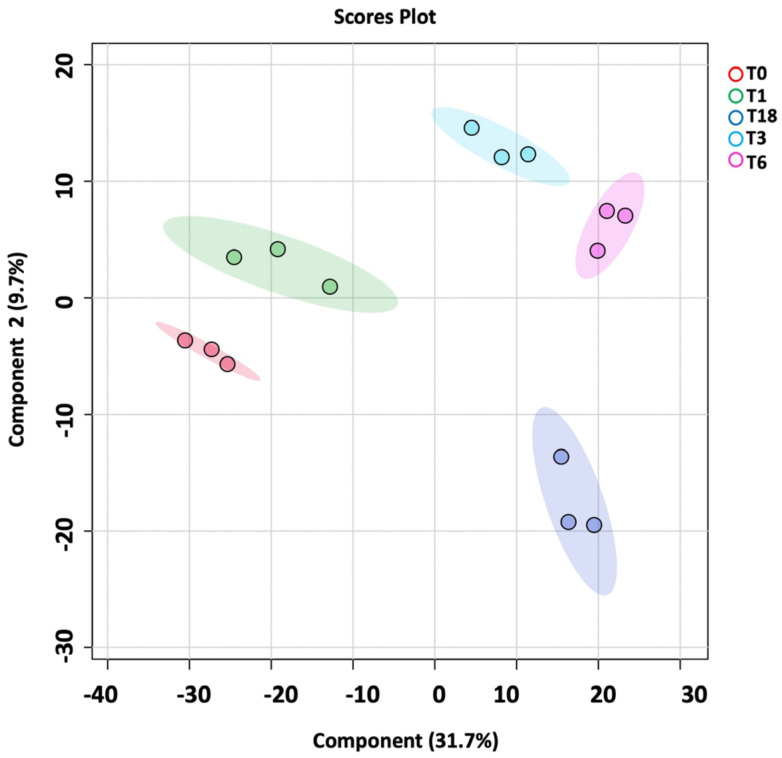
PLS-DA plot showing sample clustering in the cell pellets of *P. aeruginosa* following treatment with MDA-MB-231-conditioned media. PLS-DA plot of *P. aeruginosa* pellet shows separation between different time points (0, 1, 3, 6, and 18 h) after MDA-MB-231-conditioned treatment.

**Figure 5:**
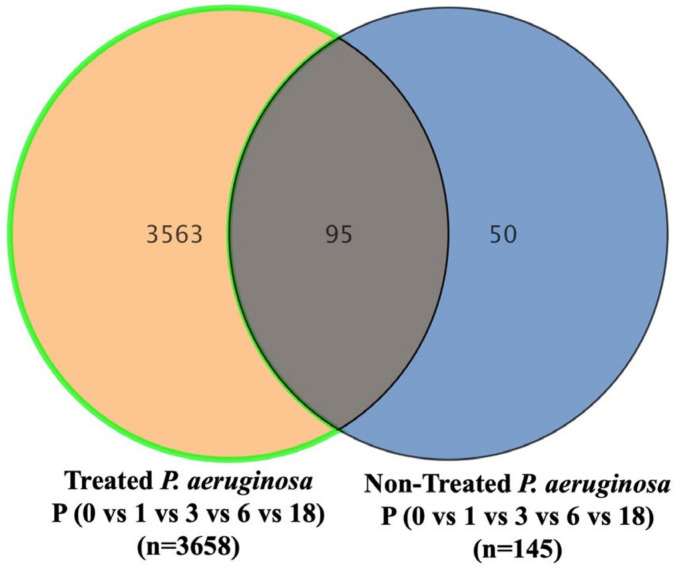
The Venn diagram showing metabolites in the cell pellets of *P. aeruginosa* following treatment with conditioned media from MDA-MB-231 or serum-free media (non-treated). The diagram shows the number of metabolites that are common or significantly dysregulated between *P. aeruginosa* treated with conditioned media from MDA-MB-231 or serum-free media. One-way ANOVA-Turkey’s post hoc FDR *p* < 0.05.

The metabolomics profile of the *P. aeruginosa* pellet and secretomes of *P. aeruginosa* treated with either BC-CM or SFM (control) were analyzed at five different time points (0, 1, 3, 6, and 18 h). First, in the *P. aeruginosa* pellet, 31,467 mass ion compounds were detected in both negative (13,449) and positive (18,018) ionization modes. The data were deposited in the Metabolomics Workbench (ST######). The detected metabolites were evaluated using partial least squares–discriminates analysis (PLS-DA), a multivariate analysis method. In this initial model, QC samples were excluded in order to focus specifically on time-dependent variations across the experimental groups. Our results show replicate clustering and a clear separation between the five time points post-treatment with BC-CM (Figure 4). To evaluate the overall data quality and model robustness, we additionally performed a separate PLS-DA analysis including QC samples, along with permutation testing. The inclusion of QC samples demonstrated proper clustering and group separation, while the permutation test validated the statistical integrity of the model, confirming that the observed separation was not due to overfitting (Figure S1A,B). After the value exclusion and imputation analysis (one-way ANOVA–Turkey’s post hoc FDR *p* < 0.05), 3658 and 145 metabolites were detected in the cell pellets of *P. aeruginosa* treated with BC-CM or SFM, respectively (Figure 5). The Venn diagram (Figure 5) shows 3563 dysregulated metabolites in the cell pellets of *P. aeruginosa* treated with BC-CM compared with the SFM-treated group that were submitted for pathway analysis (Figure S2).

Next, we used the *Pseudomonas aeruginosa* Metabolome Database (PAMDB) and Human Metabolome Database (HMDB) to assess *P. aeruginosa*-specific metabolites in the secretomes and differentiate them from those related to BC-CM. A total of 1412 metabolites out of 3563 were identified in *P. aeruginosa* secretomes after excluding exogenous molecules such as drugs, drug metabolites, and environmental exposures (Table S2). Only 262 of the 1412 metabolites were identified as endogenous metabolites (Table S3). The Venn diagram (Figure S3A) shows 313 dysregulated metabolites in the secretomes of *P. aeruginosa* treated with BC-CM compared with the SFM-treated group. The results showed replicate clustering and separation between the five time points post-treatment with BC-CM (Figure S3B), and the dysregulated metabolites were retained for further pathway analysis (Figure S3C). A moderated *t*-test (*p*-value cut-off ≤ 0.05 and fold change 2) was utilized to identify the significantly altered features between the two groups. Interestingly, the assessment between 0 and 3 h revealed 33 significantly dysregulated metabolites, where 12 metabolites were upregulated and 21 were downregulated (Table S4). Three of the upregulated metabolites, namely, citrulline, NAD, and Ubiquinol-4, and six of the downregulated metabolites, namely, PC(22:6(5Z,7Z,10Z,13Z,16Z,19Z)-OH(4)/14:1(9Z)), PA(2:0/20:5(7Z,9Z,11E,13E,17Z)-3OH(5,6,15)), PGP(18:3(9Z,12Z,15Z)/20:5(7Z,9Z,11E,13E,17Z)-3OH(5,6,15)), PIP2(16:0/16:0), citric acid, and Riboflavin, were found to be significant in *P. aeruginosa* treated with BM-CM for 3 h compared to the untreated group. These metabolites were previously identified to have essential roles in breast cancer cells (Table 1).

Similarly, the comparison between *P. aeruginosa* treated with BM-CM for 6 h and the untreated group identified 13 significantly dysregulated metabolites, with 6 metabolites being upregulated and 7 being downregulated (Table S5). Only four out of the thirteen metabolites were significant, with heme O being upregulated, while the other three metabolites, namely, uridine diphosphate glucuronic acid, citrulline, and decarboxy-SAM, were downregulated. These metabolites were identified to have a potential association with and affect breast cancer cells (Table 2).

**Figure 6:**
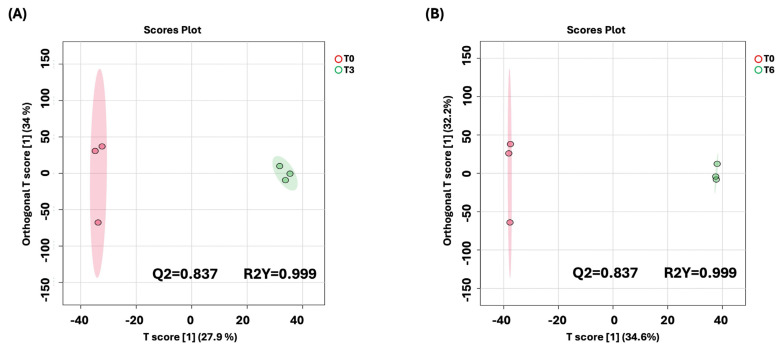
OPLS-DA model shows evident separation of BC-CM-treated *P. aeruginosa* between 0 h and 6 h. The score plot shows distinct separation for the 0 h vs. (**A**) 3 h and (**B**) 6 h samples. The robustness of the created models was evaluated by the fitness of the model (R2Y) and predictive ability (Q2) values in a larger dataset (*n* = 100).

To better understand the time-dependent metabolic changes following BC-CM treatment, we performed an OPLS-DA analysis comparing *P. aeruginosa* at 0 h vs. 3 h and at 0 h vs. 6 h post-treatment. The score plot (Figure 6 A,B) revealed a clear separation between the two time points, indicating significant metabolic shifts over time. The model’s quality was evaluated using R^2^Y and Q^2^ values, which confirmed that the observed separation was statistically robust and that the model had strong predictive performance.

Importantly, upon treatment with the conditioned media of MDA-MB-231, *P. aeruginosa* produce multiple metabolites as virulence factors (Table 3), which are reported to influence cancer cell proliferation and survival. Demonstrating the impact of BC-CM on the metabolomic profile of *P. aeruginosa,* along with the previously reported reciprocal effect of the conditioned media from bacteria on BC cells, suggests a crosstalk between the two that could contribute to the disease progression.

## 3. Discussion

Breast cancer (BC) is the most common malignancy in women [2]. Genetic anomalies in BC cells alter their metabolism to maintain cell growth, energy, and survival over time. The impact of BC cells on the microbiome present in the BC microenvironment has not yet been elucidated. This study focuses on understanding the relationships between cancer cells and microbial communities to comprehend how compounds from BC cells impact the metabolism of the breast bacterial microbiome.

Our results indicate that MDA-MB-231-conditioned media significantly distorted the *P. aeruginosa* growth rate, cell structure, and biofilm formation. When exposed to MDA-MB-231 CM (15% and 20%), *P. aeruginosa* showed a continuous slow growth followed by a nonstop exponential phase. This could be due to the formation of transformed bacterial cells known as persister cells, which are a small fraction of quiescent bacterial cells that survive lethal antibiotics or environmental stresses (L Lewis 2005). The structure of *P. aeruginosa* cells is seriously harmed by the secretions of cancer cells, which manifest through the swelling of the cell walls and the development of apparent pores on the outer membrane that allow cellular material to leak out. While our findings demonstrate significant metabolic alterations in *P. aeruginosa* upon exposure to breast cancer-conditioned media, any suggestion that these bacterial metabolic changes might influence cancer cell adaptation or therapeutic resistance remains speculative and requires further experimental validation through co-culture studies and functional assays.

Eight related human cells’ metabolites were significantly induced, which may affect *P. aeruginosa* metabolism. Iron citrate, which is metabolized in the reverse Krebs cycle through reductive carboxylation from glutamine, was significantly upregulated during the metabolic profiling of MDA-MB-231 cell excretions. The increased uptake of glutamine is linked to cancer cell growth and metabolism via the production of citrate through the reverse Krebs cycle, which provides essential metabolic substrates that support cancer cell proliferation [46]. Citrate is also known to play a crucial role in bacterial physiology, with its high levels providing *P. aeruginosa* with iron and inhibiting its synthesis of pyoverdine, thus affecting *P. aeruginosa* growth and pathogenicity [47,48].

Furthermore, this study found an increase in nicotinamide adenine dinucleotide (NAD) levels in MDA-MB-231 CM, a molecule found in all organisms [49]. NAD+-dependent signaling pathways include enzyme regulation, DNA repair, transcription, apoptosis, and metabolism. CDP-ethanolamine, a crucial metabolite involved in phosphatidylethanolamine (PE) synthesis, was also upregulated in MDA-MB-231 CM. These findings suggest a shift in membrane composition that may support cancer cell survival and functionality during stress [50,51]. PE is also important for the functional modification of bacterial membrane proteins, indicating its broader metabolic significance beyond cancer cells [52].

The metabolic profile of MDA-MB-231 CM comprises specific lipids and phospholipid-related metabolites. Pathogenic bacteria can exploit the host’s cell lipid metabolism and employ those molecules as building blocks and energy sources for their metabolic processes [53]. Each bacterial species possesses distinct metabolic capabilities within its genome, thus rendering bacterial metabolism dynamic and adaptable to the available nutrients and physical conditions in complex environments or cells for bacterial survival and pathogenicity [54].

When exposed to MDA-MB-231 CM, *P. aeruginosa* significantly upregulates purine and FR901464 metabolism. Purines are essential components of DNA and RNA and are also components of many bacterial coenzymes [55]. FR901464 is an organic bacterial product and has potent anti-proliferative and cytotoxic effects on cancer cells, including BC cells. *P. aeruginosa* naturally produce FR901464, a natural product for cancer treatment [40,41].

L-Alanine-D-glutamate-meso-2,6-diaminoheptanedioate-D-alanine, N-Acetyl-D-Glucosamine 6-Phosphate (GlcNAc-6-P), and farnesyl pyrophosphate (FPP) synthetases are also significantly increased by *P. aeruginosa* incubated with BC-CM. These metabolites play crucial roles in the cell wall biosynthesis and are essential for the survival of pathogenic bacteria [56,57]. Phosphoribosyl-AMP, an intermediate in histidine biosynthesis, was upregulated in *P. aeruginosa* under the influence of BC cell secretions [58,59].

Furthermore, our data reveal that the production of guanosine monophosphate metabolism was enhanced in *P. aeruginosa* via BC cell secretions, which are key effectors in cell adhesion and prime bacterial cell interactions with other cells and surfaces [45,60].

We also showed that the 2-aminoacetophenone (2-AA) was activated by BC excretions, which may, in turn, allow *P. aeruginosa* to survive and form persistent cells. High levels of 2-AA in *P. aeruginosa* encourage persistent cell formation within host cells by altering the metabolic pathways in immune cells to their advantage [44].

Our study showed that the *P. aeruginosa* affected by BC-CM exhibited significant induction of pyochelin I, a primary siderophore produced by *P. aeruginosa* for iron absorption [42]. Pathogenic bacteria often use siderophores as virulence factors during host infections, binding to iron found in the extracellular medium and transporting it into bacterial cells via specific outer membrane transporters, thus leading to the effective inhibition of infected host cell proliferation [42,43]. Siderophores can also be used to deliver toxic metals such as copper and gallium to the vicinity of cancer cells, resulting in reduced cell proliferation or cell death [43].

Here, we demonstrated that BC cells’ secretions influence *P. aeruginosa* to produce specific lipids and phospholipid-related metabolites, which are crucial for bacterial cell structure and separation from the environment [61]. Nine deregulated metabolites in *P. aeruginosa* exposed to BC-CM play key roles in guiding cell growth and survival. Two of these metabolites, PC(22:6(5Z,7Z,10Z,13Z,16Z,19Z)-OH(4)/14:1(9Z)) and PA(2:0/20:5(7Z,9Z,11E,13E,17Z)-3OH(5,6,15)), were downregulated and may support cancer cell proliferation by reducing the lipid signaling pathway activity [26,27]. In the presence of the BC cell secretions, *P. aeruginosa* increases NAD levels, which could alter the signaling pathways in both cancer and bacterial cells [34]. The direct effects of these changes on tumor immunity or angiogenesis were not experimentally evaluated in this study, despite the fact that our metabolomic profiling showed notable changes in NAD and citrulline metabolism in *P. aeruginosa* exposed to breast cancer-conditioned media. Functional assays, such as co-culture systems with immune or endothelial cells, should be used in future research to directly test whether these bacterial metabolic changes affect angiogenic pathways or host antitumor immunity.

Specific *P. aeruginosa* metabolites can either boost or undermine mitochondrial functions, shaping the environment for tumor growth. The downregulation of decarboxy-SAM, which is essential for polyamine production, indicates a potential vulnerability in mitochondrial integrity and cell proliferation. Polyamines are crucial for cellular functions, and their reduction could impair energy production, making cancer cells more susceptible to therapeutic interventions [39]. On the other hand, the upregulation of heme O suggests a compensatory mechanism to meet the energy demands of proliferating cancer cells, thus further complicating the metabolic picture [37,38].

The downregulation of uridine diphosphate glucuronic acid suggests a reduction in hyaluronic acid production, which could have profound implications for the cell signaling pathways crucial for BC progression. By highlighting this potential vulnerability, our results suggest that targeting these metabolic pathways could be a strategic approach to disrupting cancer cell adaptation to their microenvironment [36]. In essence, our study sheds light on the dual roles of *P. aeruginosa* metabolites in cancer biology, revealing how their fluctuations can dictate the fate of tumor cells. Understanding these metabolic alterations not only enhances our comprehension of cancer metabolism but also lays the groundwork for developing innovative therapeutic strategies that could exploit these vulnerabilities.

This study identified certain metabolomic pathway alterations (oncogenic and tumor suppressor metabolites) after exposure to breast cancer cells. It explains how cancer cells stimulate bacteria to persist and transform into cancerous bacteria that thrive in advanced tumors. The interaction between BC cells and *P. aeruginosa* presents a novel perspective on how cancer cells can influence microbial behavior. The findings suggest that cancer cells could support the survival and virulence of *P. aeruginosa,* creating a microenvironment with mutually reinforcing cellular interactions that foster both cancer progression and bacterial pathogenicity. This interplay emphasizes the importance of exploring cancer–microbiome interactions further, as such insights could lead to transformative strategies in precision oncology. The potential reciprocal effects of altered bacterial metabolism on cancer cell behavior represent an important area for future investigation, rather than merely a conclusion from our current data. Overall, our work opens new avenues for research into how manipulating metabolic pathways could yield therapeutic benefits, ultimately contributing to more effective cancer treatments.

## 4. Methodology

### 4.1. Breast Cancer-Conditioned Media Preparation

The breast cancer cell line (MDA-MB-231) was purchased from ATCC (Manassas, VA, USA) and was maintained in complete media comprising DMEM, 10% fetal bovine serum, 200 mM L-glutamine, and antibiotic–antimycotic liquid (all from Invitrogen, Paisley, UK). The cells were kept at 37 °C in 5% CO_2_ humidified tumorsphere and were routinely screened for mycoplasma using a PCR-based kit from iNtRON (Seongnam-si, Republic of Korea). Once the cells reached ~80% confluence, the complete media were removed, and the cells were washed with PBS and then incubated in serum-free media (SFM) for 24 h [62]. The cell-free breast cancer-conditioned media (BC-CM) were collected and centrifuged at 2000 rpm for 5 min. The BC-CM were then filtered using a 0.4 μm filter and stored at −80 °C until use [63].

### 4.2. Bacterial Culture and Growth Assessment by Optical Density

The *P. aeruginosa* (ATCC 27853) were cultured on solid agar. After 24 h, colonies were selected and inoculated in triplicates in 100 mL of nutrient broth (Sigma-Aldrich -Merck KGaA, Darmstadt, Germany) supplemented with either SFM (control) or increased concentrations (0%, 1%, 15%, and 20%) of BC-CM and incubated at 37 °C on an orbital shaker at 160 rpm, as previously described [17]. Bacterial growth was assessed at 0, 1, 6, and 24 h of incubation by measuring the culture’s optical density (OD) at 600 nm using a spectrophotometer (Libra S22, Biochrom Ltd., Cambridge, UK) [18]. The OD method was selected due to its convenience for frequent sampling from multiple conditions and compatibility with downstream metabolomic workflows, the main aim of our study.

### 4.3. Morphology Analysis by Scanning Electron Microscopy (SEM)

Scanning electron microscopy (SEM) was used to observe the *P. aeruginosa* morphology that were treated with 0%, 1%, 15%, and 20% of BC-CM. The samples were collected after 6 h of incubation and were fixed separately in ice-cold 2.5% glutaraldehyde in 0.05 M sodium cacodylate buffer (SCB) for 2 h to preserve their ultrastructure. The bacterial cells were washed three times with SCB and then fixed in 1% osmium tetroxide for 2 h. The bacterial samples were dehydrated through a series of ethanol solutions and treated with hexamethyldisilazane for further dehydration to minimize cellular shrinking. Finally, the samples were sputter-coated with gold particles using the Emitech K575X Sputter Covering Unit and then examined using a FEI Quanta 3D field emission gun (FEG) dual-beam SEM at 5 kV [64].

### 4.4. Preparation of Metabolite Extracts from the Cells and Supernatants (Secretomes)

The bacterial culture was treated and collected at the previously indicated time points to be centrifuged at 3000 rpm for 10 min. The cell pellets were used to extract intracellular metabolites, while the supernatants (secretomes) were filtered and collected into a 50 mL tube for extracellular metabolite analysis [65]. For intracellular metabolite extraction, bacterial cell pellets were washed with cold 1x PBS, vortexed, and spun down at 16,000 rpm at 4 °C for 10 min. The supernatants were discarded before adding 1 mL of extraction solvent (acetonitrile, methanol, and water) (ACN: MeOH: H_2_O) at a ratio of 40:40:20 to the cell pellets. Lastly, the samples were vortexed, dried overnight, and stored at −80 °C for LC-MS analysis. Finally, 900 μL of the extraction solvent at 50% (ACN: MeOH) was added to 100 μL of filtered secretomes for extracellular metabolites analysis. The samples were vortexed in a thermomixer for 1 h, centrifuged (Christ, Germany) at 16,000 rpm for 10 min at 4 °C, and then dried overnight in a speed vac (SpeedVac; Christ, Germany) and stored at −80 °C for LC-MS analysis.

### 4.5. Metabolomics Analysis

#### LC-MS Metabolomics

The dried samples were reconstituted in 50% mobile phases A (A: 0.1% formic acid in dH_2_O) and B (0.1% formic acid in 50% MeOH and ACN) for LC-MS metabolomics analysis. Additionally, 5 μL of the sample was injected into the ACQUITY UPLC XSelect column (100 × 2.1 mm × 2.5 μm) (Waters Ltd., Elstree, UK), where polar compounds were separated in reversed-phase mode using a mobile phase flow rate of 300 μL/min. Mobile phases A and B were injected into a column in a gradient mode as follows: 95–5% A (0–16 min), 5% A (16–19 min), 5–95% A (19–20 min), and 5–95% A (20–22 min). This was followed by an electrospray ionization (ESI) source to either positively or negatively ionize the LC-eluted molecules. The ESI source temperature and capillary voltages were adjusted. Both ionization modes set the source desolvation and cone gas flows at 800.0 L/h and 50 L/h, respectively. The ionized gas phase molecules were separated based on their *m*/*z* using high-resolution Quadrupole time-of-flight mass spectrometry (Xevo G2-S QTOF, Waters Ltd., Elstree, UK). The collision energy of low and high functions was set at 0 and 10–50 V, respectively, in MS^E^ (Data Independent Acquisition (DIA)) mode. The mass spectrometer was calibrated, as recommended by the vendor, using sodium formate in the range of 100–1200 Da in both ionization modes. The lock mass compound, leucine-enkephalin (an external reference to the ion *m*/*z* 556.2771 in (ESI+) and 554.2615 (ESI-)), was continuously injected by switching between a sample and reference every 45 and 60 s for ESI+ and ESI−, respectively. The lockspray was infused for 0.5 s at a flow rate of 10 μL/min, with the cone voltage set at 30 V and the collision energy at 4 V. The samples were acquired, and the DIA data were collected in continuum mode using MasslynxTM V4.1 (Waters Inc., Milford, MA, USA). Pooled QC samples were prepared using the same sample process and were introduced to the instrument after each of the ten random study samples to check for system stability [66], with the acceptance criteria requiring all QC samples to cluster together, separate from other study groups, and maintain an RSD% < 40%.

### 4.6. Data and Statistical Analysis

The MS raw data were processed following a standard pipeline starting from alignment based on the *m*/*z* value and the ion signals’ retention time, followed by peak picking and signal filtering based on the peak quality using the Progenesis QI v.3.0 software from Waters (Waters Technologies, Milford, MA, USA). MetaboAnalyst version 5.0 (McGill University, Montreal, QC, Canada) (http://www.metaboanalyst.ca, accessed on 25 March 2024) was used for multivariate statistical analysis [67]. For proper selection, in the right statistical model, the datasets (compounds and raw abundances) were mean normalized, Pareto scaled, and log transformed to maintain their normal distribution. The normalized datasets were used to generate the partial least squares–discriminant analysis (PLS-DA) model. The created OPLS-DA models were evaluated using the fitness of the model (R2Y) and predictive ability (Q2) values [68]. Univariate analysis was performed using the Mass Profiler Professional (MPP) software v15.0 (Agilent, Santa Clara, CA, USA). One-way analysis of variance (ANOVA) with Tukey’s post hoc analysis was performed for the different time points, where the features considered significant at false discovery rates displayed corrected *p*-values (FDR) *p* < 0.05. Venn diagrams were developed using MPP Software v15.0 (Agilent Inc, Santa Clara, CA, USA) [69]. According to the Pearson similarity test, heatmap analysis for altered features was performed using Pearson’s distance measure.

### 4.7. Metabolites Identification

The significant features in each dataset were selected and tagged in Progenesis QI software v.3.0 (Waters Technologies, Milford, MA, USA) for peak annotation. The chemical structures of metabolites were determined by acquiring their accurate precursor masses, fragmentation pattern, and isotopic distributions from the Human Metabolome Database (HMDB) and *P. aeruginosa* Metabolome Database (PAMDB). Exogenous compounds, including food additives, drugs, and environmental compounds, were excluded from the final list.

## Figures and Tables

**Table 1:** Significantly altered metabolites in *P. aeruginosa* treated with BC-CM (0 vs. 3 h) and their known impact on breast cancer cells.

Compound Name	*p* Value [3] vs. [0]	FC * [3] vs. [0]	Log FC [3] vs. [0]	Regulation [3] vs. [0]	Effect on Breast Cancer
PC(22:6(5Z,7Z,10Z,13Z,16Z,19Z)-OH(4)/14:1(9Z))	<0.0005	16	−4	down	Affects cell proliferation through membrane and lipid signaling [26].
PA(2:0/20:5(7Z,9Z,11E,13E,17Z)-3OH(5,6,15))	<0.0005	16	−4	down	Enhances cell survival and proliferation via mTOR activation [27].
Citrulline	<0.0005	4.9266753	2.3006144	up	Involved in NO production, promoting angiogenesis, immune suppression, and inflammation [28].
PGP(18:3(9Z,12Z,15Z)/ 20:5(7Z,9Z,11E,13E,17Z)-3OH(5,6,15))	<0.0005	16	−4	down	Precursor of cardiolipin, linked to cancer progression [29].
PIP2(16:0/16:0)	<0.0005	16	−4	down	PI3K activation supports cell growth and resistance to apoptosis [30].
Citric acid	<0.0005	16	−4	down	Involved in TCA cycle; can induce cell cycle arrest and promote apoptosis [31,32].
Riboflavin	<0.0005	16	−4	down	Mixed effects; can inhibit or promote cell proliferation and invasion at high doses [33].
NAD	<0.0005	4.3397436	2.1176097	up	Elevated NAD⁺ levels support the metabolic demands of proliferating cancer cells [34].
Ubiquinol-4	<0.0005	2.0339475	1.0242825	up	Enhances mitochondrial efficiency, supporting cell metabolism and growth [35].

* The fold change values shown as “16” represent the upper limit set by the analysis software. In some cases, the actual fold changes may have exceeded this value, but they were capped automatically due to software-imposed thresholding.

**Table 2:** Significantly altered metabolites in *P. aeruginosa* treated with MDA-231-MB-conditioned media (0 vs. 6 Hours) and their impact on breast cancer cells.

Compound Name	*p* Value [6] vs. [0]	FC [6] vs. [0]	Log FC [6] vs. [0]	Regulation [6] vs. [0]	Effect on Breast Cancer
Uridine diphosphate glucuronic acid	<0.0001	14.774896	−3.885076	down	Promotes tumor growth and metastasis via increased hyaluronic acid production [36].
Citrulline	<0.0001	16	−4	down	Influences NO production, angiogenesis, immune suppression, and chronic inflammation in cancer [28].
Heme O	<0.0001	4.720601	2.2389705	up	Enhances mitochondrial respiration and supports the metabolic needs of cancer cells [37,38].
Decarboxy-SAM	<0.0001	15.084385	−3.914984	down	Regulates polyamine biosynthesis, promoting tumor growth, invasion, and metastasis [39].

**Table 3:** Metabolites associated with virulence factors in *P. aeruginosa* after treatment with MDA-MB-231-conditioned media.

Metabolite Name	*p*-Value	Regulation	Role
FR901464 cytotoxic	0.002690988	Up	Anti-proliferation effect on cancer cells due to its ability to regulate the splicing process [40,41].
Pyochelin I siderophore	0.030434728	Up	Inhibits cancer cells’ proliferation or leads to cancer cell death [42,43].
2-aminoacetophenone	<0.0001	Up	Able to encourage persistent cells formation within host cells by altering the metabolic pathways in immune cells to their advantage [44].
Guanosine monophosphate	0.018803272	Up	Affects cell–cell/cell–surface interactions in biofilm formation [45].

## Data Availability

All data generated in this study are included in this published article (and its Supplementary Materials File) and are otherwise available from the corresponding author upon reasonable request.

## Supplementary Materials

The following supporting information can be downloaded at: https://www.mdpi.com/article/10.3390/ijms26115003/s1.
