# Supplementary material for: The MDA-MB-231 Breast Cancer Cell Secretomes Modify Metabolomes of Pseudomonas aeruginosa Breast Microbiome
Source: Int J Mol Sci. 2025 May 22;26(11):5003. doi: 10.3390/ijms26115003 (PMC12155445; doi:10.3390/ijms26115003)
Supplement: Supplementary file 1 [file ijms-26-05003-s001.zip › ijms-3613431-supplementary.pdf]

Supplementary Materials: The following supporting information can be downloaded at https://www.dropbox.com/scl/fo/3c8k19hh96d2o7a108g94/AIMROgdqCnE7C4_xSDPn40k?rlkey=mygannl82e39ftf12ty8wmamt&st=ymv5fmvo&dl=0. Figure S1: PLS-DA plot analysis of treated *P. aeruginosa* and QCs. A) PLS-DA plot shows separation between study group at different time points and QCs. B) PLS-DA permutation test. Figure S2: Pathway Analysis of the dysregulated intracellular metabolites in *P. aeruginosa* cells pre-and post-treatment with MDA-MB-231 secretomes at different time points. Variation in colors (yellow to red) indicates increase in the significance level of the dysregulated metabolites. Figure S3: Dysregulated extracellular metabolites in *P. aeruginosa* cells pre-and post-treatment with MDA-MB-231 secretome at different time points. A) Venn diagram shows the relation between significantly dysregulated extracellular ions in *P. aeruginosa* treated with MDA-MB-231 secretome (n = 354) and non-treated cells (n = 559) at five different time points (0, 1, 3, 6, and 18 hrs.). B) PLS-DA plot represents the separation of *P. aeruginosa* post-treatment samples at five different time points (0, 1, 3, 6, and 18 hrs.) based on selected 313 extracellular metabolites. C) Pathway Analysis for the significantly dysregulated metabolites that are secreted by *P. aeruginosa* after treatment with MDA-MB-231 cell secretome. 22 metabolites were ultimately identified as *P. aeruginosa* endogenous metabolites.Table S1: MDA-MB-231-related excreted metabolites; Table S2: One-way ANOVA for *P. aeruginosa* treated with MDA-MB-231 secretions after background exclusion (Intracellular samples); Table S3: Significantly dysregulated endogenous intracellular metabolites of *P. aeruginosa* treated with MDA-MB-231 cells secretions; Table S4: Comparison of significantly altered metabolites in *P. aeruginosa* treated with MDA-MB-231 CM between time points 0 and 3 hours.; Table S5: Comparison of significantly altered metabolites in *P. aeruginosa* treated with MDA-MB-231 CM between time points 0 and 6 hours.
